# Drama-based education to motivate participation in substance abuse prevention

**DOI:** 10.1186/1747-597X-2-11

**Published:** 2007-04-05

**Authors:** Aileen B Stephens-Hernandez, Jonathan N Livingston, Karen Dacons-Brock, Howard L Craft, Amura Cameron, Steven O Franklin, Allyn C Howlett

**Affiliations:** 1 Neuroscience of Drug Abuse Research Program, Julius L. Chambers Biomedical/Biotechnology Research Institute EXPORT Center, North Carolina Central University, Durham, NC, 27707 USA; 2 Department of Psychology, North Carolina Central University, Durham, NC, 27707 USA; 3 Department of Theatre, North Carolina Central University, Durham, NC, 27707 USA; 4 Dept. Physiology and Pharmacology, Wake Forest University Health Sciences, Winston-Salem, NC 27157, USA

## Abstract

**Background:**

The substance abuse prevention goal of the theatre production "TUNNELS" was to provide community education on substance abuse to an audience in Durham, NC and surrounding communities. The education effort intended to increase awareness and understanding of the risk and protective factors associated with alcohol and other drug use, and to promote pro-active behaviors in substance abuse prevention within the adult community. It was hypothesized that community-based education via drama would change attitudes toward alcohol and substance abuse, and increase participation in family and community activities aimed at substance abuse prevention.

**Methods:**

A focus group comprised of educators, substance abuse researchers and local substance abuse counselors developed "life stories" of users of alcohol and other drugs and a local playwright incorporated these and other experiences into a series of six vignettes. The production was publicized throughout the Durham area, and 700 adults attending the play signed a consent form and completed the pre-play survey. The participant pool was restricted to those adults who completed both the time-1 and time-2 surveys and resided within Durham and surrounding communities. Paired comparisons of mean responses were analyzed using a paired sample two-tailed t-test. A telephone survey three months after the play assessed attitudes toward substance abuse as a disease, and whether the respondents had increased their participation in prevention activities including discussions of the play with others.

**Results:**

Viewing the play increased the knowledge base of participants regarding substance abuse as a disease, even though the audience demonstrated an appreciation of risk and protective factors prior to attending the performance. In the pre-play survey, participants indicated a strong opinion that parental involvement in teen life was important, and therefore this was not increased as a result of viewing the play. It was found that the drama increased intent to participate in substance abuse prevention activities at home and in the community. Follow-up surveys performed three months after the performance indicated that participants had discussed the play with others and had increased their participation in substance abuse prevention activities, particularly regarding donations of money.

**Conclusion:**

Drama incorporates a component of emotional response to the informational content, and the combination of emotion and information works together to promote individual intentions to become more involved in family and community prevention activities. This study demonstrates the efficacy of drama as a mechanism to educate and motivate. Support for this mechanism is warranted at the level of state, local community, school district, and faith-based and community organizations.

## Background

The allure of using theatre as an educational device naturally stems from the art form's inherent ability to engage and entertain audiences. Theatre's role in medieval Europe emerged from ritual in the Christian church [1], to one in which re-enactments of biblical events were used to teach the scriptures to the attending public in a way that these congregations would more likely remember them [2]. Today, the use of drama as a teaching device in health education continues to be explored and documented [3-6]. In the education of substance abuse prevention principles, the use of drama has been shown to be both educational and motivational [7,8]. The present study examined the effectiveness of theatre in delivering alcohol and substance abuse prevention education to self-selected members of the Durham area and surrounding communities. The study further determined whether behavioral changes leading to increased awareness, communication, and participation could be facilitated by the drama mechanism.

Presenting a theatre production that will teach as well as entertain involves careful planning. Drama cannot simply teach: an uninteresting play defeats the purpose of its presentation. In the present project, a play script that contains the targeted information was developed with the criteria that it must be entertaining. The information communicated in TUNNELS, a play about drug abuse, was developed by a group that included neuroscience and drug abuse faculty members, and drug abuse counselors and advocates from the Durham community. Recovering drug abusers, a minister, a psychologist, the theatre director, and the playwright who independently conducted his own research on the subject, were also involved in the effort.

The outreach goals of the theatre production were to stimulate awareness, communication, and community activism in substance abuse prevention. It was hypothesized that: 1. the impact of drama can change attitudes toward substance abuse; and 2. the impact of drama can encourage community involvement in substance abuse prevention activities. To test these hypotheses, a pre/post-drama test design measured the play's effectiveness in communicating information and the play's impact on changing attitudes toward drug abuse and intended involvement in substance abuse prevention activities in the community. A long-term follow-up study determined whether the drama performance had generated discussion among family and friends, and had actually stimulated participation in and support of substance abuse prevention activities.

This study provides evidence that drama can be an effective mechanism to educate and motivate. This evidence can be used to support incorporation of drama and other entertainment modes of substance abuse education into prevention activities at the level of state and local government, school district, and faith-based and community organizations.

### Community characteristics

Durham, NC, USA and surrounding communities comprised the target population for this study. Demographic data on the target population were obtained from the U.S. Census Bureau, 2000 [9]. The ethnic makeup of the Durham area, defined by designated zip-codes, consisted of: 50% White; 40% African-American; 4% Hispanic; 3% Asian; 2% Pacific Islander; 1% American Indian; and other (mixed race). The percentage of family households (married couples) represented 42% of the population. There were 14% of households headed by a female with no husband, and 4% of households headed by a male with no wife. Single-person households were 30%, with 16% being female and 12% male. The majority of adults who were raising children in the targeted area ranged in age from 25 to 54. The average size of these families (couples with children) was four persons, and the majority of the children in these families were between the ages of 6 and 11.

The target population of Durham and surrounding communities was diverse with respect to educational level, with males and females, respectively, reported as 1.1%, 1.6%: no schooling to 4^th ^grade education; 17%, 14%: 8^th ^grade or some high school; 19%, 20%: high school graduate; 21%, 25%: some college or associate degree; 21%, 23%: bachelors degree; 9%, 11%: masters degree; and 10%, 5%: professional school or doctorate degree. The annual household income in the Durham community was diverse: 28%: <$25,000; 29%: $25,000 – $50,000; 30%: $50,000 – $100,000; and 9%: > $100,000. Only about 2% of the total population was on public assistance, but 14% were below poverty level.

The play portrayed scenes directly related to the local and regional substance abuse problems. The predominant regional drug abuse problems were identified from the North Carolina Treatment Outcomes and Program Performance System Annual Report for July 2003–June 2004 [10], for those individuals admitted to substance abuse treatment programs in the state (predominantly outpatient counseling, opioid maintenance, and case management). The primary substance problems were reported to be alcohol (30%), marijuana (20%), heroin (9%), other opiates (20%), and cocaine/crack (19%). The age range of clients was under 20 (15%), 21–30 (28%), 31–40 (29%), 41–50 (21%), and 59% were male, 41% female. Special populations identified were maternal/pregnant (10%) and child/adolescent (8%), and 31% of clients were under criminal justice supervision upon admission to treatment.

Criminal justice involvement with substance abuse treatment and outcomes is an important consideration for the local community. According to the "Treatment Alternatives for Safe Communities, Criminal Justice Management" system reports of court-ordered or other criminal justice referrals [11], 75% of individuals under treatment were male, and 52% were African American. The primary substance abuse problem for these clients was alcohol (32%), marijuana (39%), and crack/cocaine (24%). On a typical 24-hour period in the summer months that the play was performed, the Durham County Sheriff's Department detention center inmate population search [12] indicated that 23% of individuals were incarcerated due to drug-related offences (driving while impaired, possession of controlled substances or paraphernalia, intoxicated and disruptive, possession with intent to sell), and 90% of these were male. Other incarcerated individuals could also have had drug involvement for their charges (murder, assault, breaking and entering, larceny).

### The learning objectives

**Figure 1:**
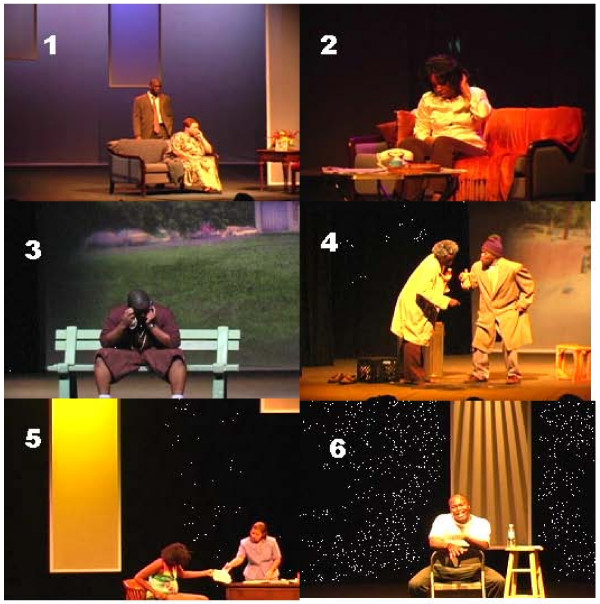
**Scenes of the Play TUNNELS^#^**. Scene 1: A mother and father try to place the blame on each other for their son losing his freedom to a robbery gone badly, that ended in murder after the victim dies of his injuries. The mother tells the father he was never there for his son, and that is why he became involved with the wrong crowd. The father tells the mother she was too soft on her son, and that it is her fault that he turned out the way he did. [See Additional File 1]. Scene 2: A woman addicted to heroin allows her drug of choice to become her driving force. In her hallucinating conversations with this powerful entity, she is convinced by it that she will never be able to break free. She allows it to ruin her career and any close relationships. [See Additional File 2]. Scene 3: A drug dealer rationalizes that he is not the problem in his community; rather, he is only providing a service to those who are going to get drugs from him or someone else. He calls himself "the poor man's version of an entrepreneur." His feeling is that his crack dealing business will always do well because "crack sells itself". He gets arrested eventually. [See Additional File 3]. Scene 4: Two men with an alcohol abuse problem have lost everything and are living on the streets. They have no idea where their families are. Their memories of them are very few. They rely on each other to keep their habit supported, until one dies while the other was away. [See Additional File 4]. Scene 5: A young pregnant woman tries to use the welfare system as a means of support for her crack addiction. She has time and time again gotten services by misrepresentation of her situation. Her social worker wants her to go into treatment, but she is reluctant. When she is left alone she steals money from the social worker. [See Additional File 5]. Scene 6: A young man is in jail for murder because he allows his girl-friend's child to be present during a crack binge, and the child inadvertently shoots his mother with the boy-friend's gun. He tries to cover it up giving the child crack. He gets stopped by the police while trying to leave town. He tells a heart-felt story of how using drugs can narrow a life to convoluted TUNNELS. [See Additional File 6].

The educational goals of the play included that the audience should understand that drug abuse is a disease. The scenes of the play TUNNELS are depicted in Fig. 1 and Additional Files [Additional File 1 to Additional File 6]. The play depicted a woman dependent on heroin, an incarcerated man whose judgment had been impaired while on a crack binge, and two men with an alcohol abuse problem engaged in the pursuit of their next drink. The audience should also know that drug use can damage many organs of the body. This was depicted in scenes of men who experienced generally poor mental and physical health as a result of chronic alcohol abuse, and a pregnant woman who was purchasing crack. The concept of drug dependence was depicted in scenes that showed heroin and alcohol withdrawal. The American Psychiatric Association Diagnostic and Statistical Manual-IV-Text Revision (DSM IV-TR) criteria for substance dependence and substance abuse [13] were depicted in all scenes as situations in which the drug, and activities related to obtaining the drug, consumed a major part of the person's life, and those who consumed drugs continued to do so in spite of negative consequences. The woman dependent on heroin considered the drug to be more important than other activities of normal life although drug use seriously jeopardized her job and relationships. The men who were dependent on alcohol had lost home, family and employment.

The negative consequences of drug use were primarily depicted as the criminal justice involvement. The crack cocaine dealer was arrested, an adolescent was in jail for a robbery involving the shooting death of a store clerk, a woman dependent on heroin and a pregnant woman stole from friends and strangers, and child neglect led to a shooting death during a crack binge. The negative consequences to family were depicted by a scene in which the parents were devastated by their son's drug crimes, a crack dealer was involving his young child in home crack production activities, two men had abandoned and neglected their families as the result of alcohol abuse, and a pregnant woman exhibited little concern for the effects of her drug use on her unborn child.

The risk factors that are associated with the development of drug problems included ineffective parenting in which a parent was too involved in career activities to participate in his son's life. The notion that children are at risk by learning their parent's behavior and living in a chaotic home environment, was depicted by a crack cocaine dealer with a young son, and a child who was a participant in crack use at home. Poor social coping skills were the subject of a discussion by parents of a son who failed to interact with high economic status peers in his new neighborhood, and a crack dealer whose grade school experience excluded him from being considered to be a successful student. The crack dealer maintained affiliations with other crack dealers, and failed to integrate into the normal work force.

The protective factors of strong family bonds and the necessity of parental monitoring and involvement were discussed in a scene by parents of a son in jail for a drug-related crime. Rewarding success in school performance was a protective factor that was lacking in the same situation, as well as in the educational environment of the crack dealer.

## Results

The play TUNNELS was performed at an Historically Black University in northern North Carolina. The audience had learned about the play from local media (newspapers and radio (12.7%), flyers that had been posted in public places (9.7%), word of mouth (37.5%) and other mechanisms (23.6%) which included postal mailings to individuals on the university events list, and announcements distributed on email groups, list-serves, and the university web site (Table 1). The sample for study was comprised of consenting adult members of the audience who identified the zip code of their residence to be Durham and surrounding communities (which totaled 67% of the adult audience).

**Table 1:** Characteristics of the adult population that attended the play

**Drama Audience Characteristics**	**Total Adult Participants^1^**	
Male	206	29.7%
Female	487	70.2%
**Age**		
20–29	149	21.2%
30–39	158	22.5%
40–49	168	23.9%
50–65	169	24.1%
Over 65	56	8.0%
**Learned about the play from:**		
Newspaper	47	6.7%
Radio	42	6.0%
Church bulletin	27	3.8%
Word of mouth	263	37.5%
Flyers and Posters	68	9.7%
Other	166	23.6%
More than one source	88	12.5%
**Totals**	711	100%

^1^Valid percentages do not incorporate missing data.

### The impact of the play TUNNELS on attitudes

It was found that individuals who voluntarily attended the play TUNNELS, which was advertised to be a play focused on substance abuse, were members of the community who appear to be interested in the subject and have relatively high level of understanding of substance abuse as a disease. The adult audience was 89% in agreement that alcohol and drug use is a disease. The majority, 75%, somewhat or strongly disagreed that alcohol and drug addicts can quit at any time, whereas 80% somewhat or strongly agreed that addicted persons must go through treatment to stop. However, 84% felt that drug use is a choice. This combination of responses might suggest that these respondents believe that substance abusers must choose to seek treatment in an effort to quit. There was 79.5% agreement that persons addicted to drugs would continue to use drugs in spite of negative consequences, and 92% agreement that life-style factors are involved in substance abuse. The adult audience were quite divided regarding their opinion on whether drug use is a character flaw, with 34% strongly disagreeing, 26% somewhat disagreeing, 23% somewhat agreeing and 17% strongly agreeing with the statement. Regarding health consequences, 89% believed that one of the negative consequences of drug use is the spread of disease.

The importance of the parental role was strongly advocated by the adult audience prior to viewing the drama. Regarding parental involvement, 97% agreed that parents need to be involved in their children's school activities, and 97.5% agreed that parents need to keep track of their teens' activities. Furthermore, 98% agreed that parents should set a good example with respect to substance use. These convictions were so strong that viewing the play could not increase them further (Table 2).

**Table 2:** Responses in the pre- and post-play surveys on knowledge and attitudes towards alcohol and substance abuse.

**Knowledge about alcohol and drug use/abuse**	**N**	**Time-1 ** X^1 ^± SD (SEM)	**Time-2 ** X ± SD (SEM)	**Paired Difference ** X ± SD (SEM)	**Significance ** 2-tailed p
Alcohol and other drug use is a disease.	226	3.52 ± 0.828 (0.055)	3.61 ± 0.754 (0.050)	+0.089 ± 0.6540 (0.0435)	0.043 *
An alcoholic or drug addict can quit at any time.	228	1.69 ± 0.930 (0.062)	1.71 ± 0.942 (0.062)	+0.013 ± 0.8829 (0.0585)	0.822
Alcohol and other drug use is a choice.	226	3.32 ± 0.825 (0.055)	3.14 ± 0.963 (0.064)	-0.181 ± 0.9418 (0.0627)	0.004 *
Persons who are addicted to alcohol or other drugs must go through treatment to stop using.	229	3.16 ± 0.884 (0.058)	3.32 ± 0.822 (0.054)	+0.166 ± 0.8210 (0.0543)	0.002 *
Alcohol and other drug use is character flaw.	216	2.21 ± 1.075 (0.073)	2.29 ± 1.162 (0.079)	+0.074 ± 1.023 (0.0696)	0.288
Persons who are addicted to alcohol or other drugs will continue to use even though there are negative consequences	221	3.25 ± 0.867 (0.058)	3.44 ± 0.782 (0.053)	+0.190 ± 0.8739 (0.0588)	0.001 *
Some lifestyle factors are associated with drug abuse problems.	226	3.46 ± 0.699 (0.047)	3.57 ± 0.637 (0.420)	+0.115 ± 0.6832 (0.0454)	0.012 *
Parents should try to be involved in their children's school activities	228	3.89 ± 0.425 (0.028)	3.92 ± 0.355 (0.024)	+0.035 ± 0.4773 (0.0316)	0.268
Parents need to set a good example in the home when it comes to drug or alcohol use.	222	3.93 ± 0.349 (0.023)	3.94 ± 0.337 (0.023)	+0.009 ± 0.3007 (0.0202)	0.656
Parents need to keep track of their teens' activities and know who their friends are.	223	3.91 ± 0.349 (0.023)	3.91 ± 0.397 (0.027)	0.000 ± 0.4245 (0.0284)	1.000

^1 ^Responses on a scale of 1 to 4 (1: Strongly disagree; 2: Somewhat disagree; 3: Somewhat agree; 4: Strongly agree) were averaged, and mean (X) ± standard deviation (SD) and standard error of the mean (SEM) are reported. Differences in response between time-2 and time-1 were used to determine statistically significant differences from zero using a paired sample two-tailed t test.

* Statistically significant difference at p < 0.05.

Comparisons of pre-drama and post-drama responses of the Durham area respondents indicated that a significant change occurred in attitudes toward the disease of substance abuse (Table 2). Very similar findings were obtained from the entire population of adult participants who viewed the play (data not shown). Mean differences between time-1 and time-2 scores significantly increased in agreement that alcohol and other drug use is a disease and that addicted persons will continue to use even though there are negative consequences. Of those respondents who had strongly or somewhat disagreed that drug use is a disease prior to the play, nearly half changed their opinion to somewhat agree (26.2%) and strongly agree (21.4%) after viewing the play. After the play, viewers were less likely to agree that alcohol and other drug use is a choice, and more likely to agree that addicted persons must go through treatment to stop using. After viewing the play, those respondents who at time-1 had disagreed with the statement that "addicted persons must go through treatment to stop" were more inclined to somewhat agree (44.9%) or strongly agree (15.9%) with the statement at time-2.

The mean response data (Table 2) indicated somewhat disagreement with the notion that a drug addict could quit at any time. This average opinion did not significantly change after viewing the play. However, among those respondents who had somewhat or strongly agreed with the statement, viewing the play influenced some viewers to somewhat disagree (29.1%) or strongly disagree (14.0%) with the statement. These findings suggest that after seeing the play, some viewers tended to be more convinced that the affected individual has to make a choice to initiate treatment of his substance abuse disease. The issue of "choice" and drug abuse was clarified in the follow-up telephone survey. In that survey, 97.2% agreed with the statement that "The first time a person tries drugs, he has a choice to do so". Of those same follow-up respondents, only 82.7% of them had somewhat or strongly agreed that "drug use is a choice", and 92.5% had somewhat or strongly agreed that "drug use is a disease" in the time-1 survey prior to viewing the play. In the three-month follow-up, 98.3% of the phone survey respondents agreed with the statement "A person who has a problem with alcohol or other drugs generally needs help in order to quit". In the follow-up survey, 92.7% disagreed that a "user is able to quit using voluntarily at any time", whereas only 77.2% of those same respondents had somewhat or strongly disagreed at time-1 with the statement that a "drug addict can quit at any time".

### The impact of the play TUNNELS on behaviors

Regarding personal activism and community empowerment in substance abuse prevention, prior to viewing the play, 77% of the audience felt that it is important to read books and articles related to drug use, and 79% indicated that they were interested in issues related to drug use and consequences. Of those adults who attended the play, 78% knew where to get information regarding substance use and abuse, and 80% indicated that they usually or always use information that they have learned regarding symptoms and consequences of drug use when talking to family and friends. However, only 30% volunteered with organizations involved in substance abuse prevention, only 42% actively gathered information or talked about substance abuse in a formal setting, and only 27% donated money to drug abuse missions or organizations.

The play generated an increased interest in reading more about the issues associated with drug and alcohol use and using that information for themselves and family members (Table 3). The increased awareness of where to obtain information may have been generated by the public service announcements in the play program as well as informational materials displayed on tables in the lobby of the theatre that had been provided by local community service organizations. Sixty percent of those who had never or sometimes been interested in issues related to drug use were persuaded to report an increased interest after viewing the play. Of those who had never or sometimes been inclined to use information they had learned about drugs when talking to their kids, the play induced 84% of them to respond that they would now use that information in talking with their kids. Three months after seeing the play, 81% of the follow-up phone survey respondents reported having talked to their family or friends about alcohol or other drug abuse. Increased interest in substance abuse related issues was evidenced by the fact that 73% of phone survey respondents reported remembering radio or television stories and 67% reported remembering newspaper or magazine articles related to alcohol or other drug abuse within the three months after seeing the play.

**Table 3:** Responses in the pre- and post-play surveys on personal activism and involvement in substance abuse prevention activities.

**Intentions to Act**	**N**	**Time-1 ** X^1 ^± SD (SEM)	**Time-2 ** X ± SD (SEM)	**Paired Difference ** X ± SD (SEM)	**Significance ** 2-tailed p
I believe that it is important to read books, newspaper and magazine articles related to alcohol and other drug use.	280	3.28 ± 0.817 (0.049)	3.45 ± 0.741 (0.044)	+0.171 ± 0.8504 (0.0508)	0.001 *
I am interested in issues related to alcohol and other drug use and their consequences.	280	3.26 ± 0.808 (0.048)	3.36 ± 0.778 (0.047)	+0.104 ± 0.9428 (0.0563)	0.067
I use information I've learned regarding symptoms and consequences of drug use when talking to my kids and other people.	278	3.26 ± 0.873 (0.052)	3.64 ± 0.652 (0.039)	+0.389 ± 0.8913 (0.0535)	<0.001 *
I know who to contact to get information on alcohol and other drugs.	272	3.26 ± 0.889 (0.054)	3.43 ± 0.798 (0.048)	+0.173 ± 0.8821 (0.0535)	0.001 *
When I receive information on how to improve my health and life, I use it.	255	3.25 ± 0.768 (0.048)	3.51 ± 0.793 (0.049)	+0.263 ± 0.9586 (0.0600)	<0.001 *
**Participation**					
I participate in groups or organizations that talk about or work on issues related to alcohol and other drug use:					
as a volunteer.	190	2.05 ± 1.012 (0.073)	2.73 ± 1.013 (0.0735)	+0.674 ± 1.078 (0.078)	<0.001 *
by talking to other individuals in my community.	201	2.41 ± 0.956 (0.067)	3.00 ± 0.949 (0.067)	+0.587 ± 1.048 (0.074)	<0.001 *
by gathering information on alcohol and other drug use and its consequences to share with others.	194	2.36 ± 0.988 (0.071)	3.10 ± 0.897 (0.064)	+0.742 ± 1.031 (0.074)	<0.001 *
by donating money to organizations targeting substance abuse prevention or treatment.	189	2.02 ± 0.967 (0.070)	2.67 ± 0.956 (0.070)	+0.651 ± 1.064 (0.077)	<0.001 *

^1 ^Responses on a scale of 1 to 4 (1: Never; 2: Sometimes; 3: Usually; 4: Always) were averaged, and mean (X) ± standard deviation (SD) and standard error of the mean (SEM) are reported. Differences in response between time-2 and time-1 were used to determine statistically significant differences from zero using a paired sample two-tailed t test.

* Statistically significant difference at p < 0.05.

It was found that three months after the play, nearly all of the follow-up respondents identified at least one scene of the play that was outstanding in their mind, but no single scene appeared to be more outstanding than the others (Table 4). Greater than 20% reported that two or more scenes were memorable, and more than 15% reported that the entire play was outstanding in their minds. The follow-up revealed that 99% of the telephone respondents agreed that scenes from the play were consistent with real life, and 78% thought that the entire play applied to real life. Most importantly, 86% of the respondents had discussed some scenes of the play with family and friends, and 54% had discussed the entire play (Table 4).

**Table 4:** Frequency of responses from the three-month follow-up survey to assess communication of the substance abuse prevention messages of the play beyond the audience.

**Scene:**	None	#1	#2	#3	#4	#5	#6	≥two	All
**Query**	Valid Percentages (N = 187)								
Which scenes in the play were the most outstanding in your mind?	0.5	12.3	12.3	14.4	11.2	8.0	4.8	20.8	15.5
Which scenes in the play apply to real-life?	1.1	3.2	1.6	2.1	1.6	0.5	1.6	10.1	78.1
Which scenes did you discuss with family or friends	14.4	2.1	4.8	3.2	2.1	1.6	2.1	16.1	53.5

The greatest impact of the drama was on personal activism. The pre- and post-play survey results indicated that the drama had stimulated respondents to want to seek and utilize knowledge regarding substance abuse, and donate money to substance abuse treatment and prevention missions (Table 3). Of those respondents who had reported at time-1 that they never or sometimes volunteered time, talked with others in the community or donated money, after viewing the play, 43% reported intentions to volunteer, 55% intended to talk about substance abuse issues, and 40% intended to donate money to organizations. The three-month follow-up survey assessed whether participants were actually engaged in efforts to address drug and alcohol abuse in their communities in the period of time after seeing the play (Table 5). Of the respondents in the follow-up survey, 66.7% and 72.7% had read articles or heard media reports on substance abuse since seeing the play, whereas only 46% of these same individuals had reported at time-1 that they usually or always gathered information. This might suggest that attending the play had raised their awareness of the issue so that they gave it more attention. Whereas 81% reported having talked to others about substance abuse in the three months after the play, only 43.2% of these same individuals had usually or always talked about substance abuse prior to seeing the play. It was found that 38% of follow-up respondents reported participating in activities in the community related to alcohol and drug abuse prevention, and 30.6% reported volunteering in substance abuse prevention activities at some time during the three months following the play. This represents no change over the pre-play response by these same individuals. However, of interest, 43% of the follow-up respondents reported having donated money to organizations involved in substance abuse prevention within the three-months after seeing the play. Only 30% of these same respondents had reported donating money prior to viewing the play and the exhibits in the lobby of the theatre. This suggests that the impact of drama had increased motivation to contribute money, perhaps due to an increased awareness of community treatment and prevention organizations.

**Table 5:** Frequency of responses from the three-month follow-up survey to assess sustained involvement in substance abuse prevention activities after viewing the play.

**Percentage of Follow-up Survey Respondants (N = 181)**						
**Time-1**				**Time-3**		

**I participate in groups or organizations that talk about or work on issues related to alcohol and other drug use:**				**In the last three months, have you:**		

**Never**	**Sometimes**	**Usually**	**Always**		**No**	**Yes**

by gathering information on alcohol and other drug use and its consequences to share with others.				heard or seen any radio or television stories related to alcohol or other drug abuse?	27.3	72.7
12.8	41.0	17.9	28.2	read any newspaper or magazine articles related to alcohol or other drug abuse?	33.3	66.7
						
by talking to other individuals in my community.				talked to your family or friends about alcohol or other drug abuse?	18.9	81.1
12.5	44.4	21.3	21.9	participated in any community activities related to preventing alcohol or other drug abuse?	60.7	39.3
						
as a volunteer.				volunteered for any activities in the community that are related to preventing alcohol or other drug abuse?		
26.8	43.0	12.1	18.1		69.4	30.6
						
by donating money to organizations targeting substance abuse prevention or treatment.				donated any money to local or national organizations that prevent or fight alcohol and drug abuse or local church-sponsored missions?		
29.3	40.8	15.6	14.3		57.0	43.0

## Discussion

One goal of the drama presentation was to deliver educational messages to members of the greater Durham community. The play was performed on the campus of a Historically Black University, and local churches were informed of the performances, thereby enriching the audience participation from local African American groups. This increases the impact of the educational effort to address issues of minority health disparities due to the greater proportion of the audience solicited from word-of-mouth publicity, particularly via the local African American churches. The life-time risk of substance abuse disorder is lower among African Americans than non-Hispanic Whites according to data analyzed from the National Comorbidity Survey [14,15]. Nevertheless, the criminal justice and health care consequences of substance abuse are disproportionately greater for African American and disadvantaged ethnic groups compared with White populations [16-20]. The play TUNNELS dealt with contemporary drug abuse issues and depicted culturally consistent scenarios in an effort to address the needs of the local community.

Our study found that the self-selected audience appeared to be well-informed on the concept of substance abuse as a disease. Members of the audience also expressed firm convictions about preventive factors such as appropriate parenting skills and the need for parental participation in children's social activities. Among those who did not appreciate the disease aspects of substance abuse prior to seeing the play, the effect of the drama appeared to be to increase understanding and interest. In a study by others, the viewers of a 13-episode weekly series on Bangladesh television indicated that they liked the drama [3]. This mechanism of educational delivery significantly increased the knowledge of television viewers regarding AIDS, childhood diseases, nutrition, family planning and other targeted health care issues [3-5]. The difference between the audience in our study versus the television viewers in these public health studies, is that play-goers must be motivated to leave their homes to attend the performance, and must have had an interest in the subject.

Live theatre can be used as a means to facilitate discussion of sensitive health care issues such as substance abuse. For example, a live musical play was performed in Chicago high schools that were considered to be at risk for substance abuse problems, and the viewing of the play was followed by discussion of relationships with peers, boyfriend/girlfriend and family, legal consequences of substance abuse, and where to go for help if needed [8]. The use of short plays for peer counseling sessions for pre-adolescents has been promoted as a means to open communication in a non-threatening way [6]. In our follow-up of play-goers three months after viewing the drama, all of the respondents reported that some part or all of the play was still memorable, and that they had discussed all or part of the play with their family and friends. This demonstrates that the impact of the drama was communicated beyond the individuals who had attended the play. The play had stimulated discussion, and therefore had served as a mechanism for distributing information to the community beyond those in the audience alone.

Increasing knowledge does not necessarily correlate with motivating behavioral changes. For our purposes, the goals of the dramatic presentation were not so much to increase knowledge as to motivate behavior toward community activism through self-empowerment. One might predict that the emotional responses engendered by viewing the drama would increase motivation to change behaviors. This has been reported for other drama-based health care education initiatives. Watching a health education television series was significantly associated with a greater percentage of women attending a health clinic or using modern contraception [3,5]. After viewing a live drama on substance abuse in high schools, approximately 18% of the adolescent viewers requested counseling, an indication of the impact that the drama had on stimulating these students toward a change in behavior [8]. Another study compared responses of adolescents to attending lectures that delivered an alcohol abuse message or viewing skits that communicated a similar content [7]. It was found that both lectures and skits were able to impact attitudes and short-term behavior compared with the control group [7]. After viewing a dramatic film, attitudes of college students towards drinking alcohol were modified depending upon whether alcohol use was coupled to scenes of its negative consequences or, alternatively, alcohol use was shown with the scenes of the negative consequences edited out [21]. In the present study, the effect of drama was highly successful in stimulating personal activism to participate in community and social activities aimed at substance abuse prevention. This response was sustained over a three-month period after the play, particularly regarding donating money to substance abuse prevention and treatment organizations. Our results suggest that the combination of emotion and information work together to promote individual intentions to become more involved in family and community prevention activities.

## Conclusion

In summary, the understanding of substance abuse as a disease and the motivation to change behaviors on the part of adults in an ethnically, economically and educationally diverse community can be influenced by the use of drama. In our study, the motivation to change was directed at community participation in substance abuse prevention including awareness of the problems and understanding of how to address them. Because alcohol and substance use and abuse can be a difficult subject to discuss with family and others, presentations from theatre or other modes of entertainment may be considered to be useful tools to facilitate communication. This study documents evidence to demonstrate the efficacy of drama as a mechanism to educate and motivate, and recommends support for this mechanism at the level of state, local community, school district, and faith-based and community organizations.

## Methods

Six performances of the play TUNNELS were offered over a two-week period. In an effort to assess the effectiveness of the play in changing attitudes and behaviors, a pre/post-test design was employed. A consent form and a survey containing 22 questions were distributed as the audience entered the theatre lobby. Participants were given ample time to fill out the time-1 survey prior to the beginning of the performance. Immediately following the performance, the audience remained in the theatre long enough to complete the time-2 survey before the announcement of door prizes. There were 711 adult participants who signed informed consent forms and completed the time-1 survey. After attending the drama presentation, the sample size of time-2 respondents was reduced to 478 participants. Time-1 and time-2 responses were analyzed from a sample that resided within a 14-zip-code area designated as "Durham and surrounding communities", thereby reducing the sample size to 283 participants. The make-up of the sample population from Durham and surrounding communities was comparable to the make-up of the total adult audience (Table 1).

A pre/post survey design was employed, in which respondents reported their opinions and behaviors in a test given before seeing the play. These questions regarding opinions and expected future behaviors were repeated immediately after viewing the play. Questions of opinion were offered response choices of Strongly disagree, Somewhat disagree, Somewhat agree, or Strongly agree and questions of behaviors were offered response choices of Never, Sometimes, Usually, or Always. In order to quantify attitudes or behaviors, the four response choices were assigned a numerical value of 1 to 4, respectively. Data for each respondent who answered the question at both time-1 and time-2 surveys are reported in Tables 2 and 3 as means and SD (N = 189 to 280). In order to assess the changes in attitudes or intentions to act that resulted from seeing the play, matched responses were compared. Differences between the initial response and the response after viewing of the play were analyzed by a paired sample two-tailed t-test (SPSS version 13 (SPSS, Inc.)). Mean differences were considered to be significantly different from zero (no change) at p < 0.05, and these values are reported in Tables 2 and 3. Frequency of response data are reported within the text to indicate the percentage of the participants that responded using the designated descriptors. These frequency measures are reported to illustrate how the statistically significant changes in mean differences came about, and these measures have not been used for statistical analyses themselves.

In an effort to assess the long-term impact of the play, a delayed time series design was conducted. A follow-up survey was administered by phone three months after the play, in order to assess the responses regarding prevention activities. There were 187 phone surveys completed, representing a 66% response rate from the Durham area sample population. The phone survey consisted of 13 statements to which the respondent could answer yes or no depending upon whether they agreed or disagreed with the statement. The data were collected as frequency of a yes or no response. In order to match the three-month follow-up responses with time-1 responses made by the same individuals, the frequency of the "No" response in the phone survey was compared retrospectively with the frequencies of the "Never" and "Sometimes" answers in the time-1 survey by those same individuals. Similarly, the frequency of the "Yes" response in the phone survey were compared retrospectively with the frequency of the"Usually" and "Always" responses from the time-1 survey. Data are reported as frequency of response in Tables 4 and 5, and statistical comparisons were not performed.

## Competing interests

HLC holds the copyright to the play TUNNELS. All other authors report no competing interests.

## Authors' contributions

ABS-H, JNL, KD-B, SOF and ACH participated in the focus group to develop and design the play content and the survey instruments. HLC wrote and KD-B directed and produced the play TUNNELS. ABS-H and ACH assessed community characteristics and learning objectives. ABS-H, AC, SOF and ACH organized community service agencies to provide educational materials and the post-play commentary associated with the production. ABS-H, AC, SOF and ACH collected and organized pre- and post-play data and the follow-up telephone surveys. ABS-H, AC and JNL performed the statistical analyses. ACH and JNL served as advisors for students ABS-H and AC. ACH was the team-leader for this project and obtained Institutional Review Board approval. ACH and ABS-H drafted, edited, and revised the manuscript, and all other authors provided commentary and modifications.

## Supplementary Material

Additional File 1 Scene 1 of TUNNELS. Parents blame each other for their son's behavior leading to a drug-related crime. Click here for file

Additional File 2 Scene 2 of TUNNELS. A woman is suffering from withdrawal in her heroin dependence. Click here for file

Additional File 3 Scene 3 of TUNNELS. A drug dealer describes the marketing and sale of crack cocaine. Click here for file

Additional File 4 Scene 4 of TUNNELS. Two homeless men discuss the impact of alcohol abuse on their relationship with family. Click here for file

Additional File 5 Scene 5 of TUNNELS. A pregnant woman, who needs money for her crack habit, steals from a social services worker. Click here for file

Additional File 6 Scene 6 of TUNNELS. A man is incarcerated for the accidental death of his partner during a crack cocaine binge. Click here for file

## References

[B1] Brockett O (1979). The Theatre: An Introduction  (Historical Edition).

[B2] Arnott P (1981). The Theatre in its Time, an Introduction.

[B3] Do MP, Kincaid DL (2006). Impact of an entertainment-education television drama on health knowledge and behavior in Bangladesh: an application of propensity score matching. J Health Commun.

[B4] Piotrow PT, Kincaid DL, Hindin MJ, Lettenmaier CL, Kuseka I, Silberman T, Zinanga A, Chikara F, Adamchak DJ, Mbizvo MT, . (1992). Changing men's attitudes and behavior: the Zimbabwe Male Motivation Project. Stud Fam Plann.

[B5] Piotrow PT, Rimon JG, Winnard K, Kincaid DL, Huntington D, Convisser J (1990). Mass media family planning promotion in three Nigerian cities. Stud Fam Plann.

[B6] Sturkie J, Cassady M (1992). Acting it out junior.

[B7] Gliksman L, Douglas RR, Smythe C (1983). The impact of a high school alcohol education program utilizing a live theatrical performance: A comparative study. J Drug Education.

[B8] Harding CG, Safer LA, Kavanagh J, Bania R, Carty H, Lisnov L, Wysockey K (1996). Using live theatre combined with role playing and discussion to examine what at-risk adolescents think about substance abuse, its consequences, and prevention. Adolescence.

[B9] US Census Bureau (2000). U S Census.

[B10] North Carolina Treatment Outcomes and Program Performance System (2004). Annual Report for July 2003-June 2004.

[B11] Treatment Alternatives for Safe Communities (2005). Criminal Justice Management System Reports.

[B12] Durham County Sheriff's Department (2005). Detention Center Inmate Population.

[B13] American Psychiatric Association (2000). Diagnostic and Statistical Manual of Mental Disorders.

[B14] Breslau J, Aguilar-Gaxiola S, Kendler KS, Su M, Williams D, Kessler RC (2006). Specifying race-ethnic differences in risk for psychiatric disorder in a USA national sample. Psychol Med.

[B15] Breslau J, Kendler KS, Su M, Gaxiola-Aguilar S, Kessler RC (2005). Lifetime risk and persistence of psychiatric disorders across ethnic groups in the United States. Psychol Med.

[B16] National Institute on Drug Abuse (2003). Drug Use Among Racial/Ethnic Minorities.

[B17] Sanders-Phillips K (2002). Factors influencing HIV/AIDS in women of color. Public Health Rep.

[B18] Iguchi MY, London JA, Forge NG, Hickman L, Fain T, Riehman K (2002). Elements of well-being affected by criminalizing the drug user. Public Health Rep.

[B19] Ramchand R, Pacula RL, Iguchi MY (2006). Racial differences in marijuana-users' risk of arrest in the United States. Drug Alcohol Depend.

[B20] National Institute on Drug Abuse: (2006). Criminal Justice/Drug Abuse Treatment Studies.

[B21] Bahk CM (1997). The impact of presence versus absence of negative consequences in dramatic portrayals of alcohol drinking. J Drug Education.

